# Performance Analysis of a Semiactive Suspension System with Particle Swarm Optimization and Fuzzy Logic Control

**DOI:** 10.1155/2014/174102

**Published:** 2014-01-16

**Authors:** Abroon Jamal Qazi, Clarence W. de Silva, Afzal Khan, Muhammad Tahir Khan

**Affiliations:** ^1^Department of Mechanical Engineering, University of Engineering and Technology, Peshawar, KPK, 25000, Pakistan; ^2^Department of Mechanical Engineering, University of British Columbia, Vancouver, BC, Canada V6T 1Z4

## Abstract

This paper uses a quarter model of an automobile having passive and semiactive suspension systems to develop a scheme for an optimal suspension controller. Semi-active suspension is preferred over passive and active suspensions with regard to optimum performance within the constraints of weight and operational cost. A fuzzy logic controller is incorporated into the semi-active suspension system. It is able to handle nonlinearities through the use of heuristic rules. Particle swarm optimization (PSO) is applied to determine the optimal gain parameters for the fuzzy logic controller, while maintaining within the normalized ranges of the controller inputs and output. The performance of resulting optimized system is compared with different systems that use various control algorithms, including a conventional passive system, choice options of feedback signals, and damping coefficient limits. Also, the optimized semi-active suspension system is evaluated for its performance in relation to variation in payload. Furthermore, the systems are compared with respect to the attributes of road handling and ride comfort. In all the simulation studies it is found that the optimized fuzzy logic controller surpasses the other types of control.

## 1. Introduction

A suspension system of an automobile improves the ride quality by absorbing shock and other external disturbances under various operating conditions. While doing so, it has to support both static and dynamic loading of the vehicle as well. The external disturbances may arise from a variety of sources such as road surface irregularities, aerodynamics forces, nonuniformity of the tire/wheel assembly [1], and even braking forces. Usually, road irregularities, ranging from potholes to random variations of the surface profile, are a major source of undesirable excitations of the vehicle body, which act through the tire-wheel assembly and the suspension system. In general, a good suspension system must provide an acceptable trade-off between a comfortable ride and good handling under a specified set of operating conditions [2].

An active suspension system requires external power to function, which results in added complexity, cost, and weight, as well as reliability problems, though it improves the controllability [3, 4]. The concept of semiactive suspension has emerged as a compromise, in order to reduce the complexity and cost while improving the ride and handling. In a semi-active suspension system, the springs of a conventional passive suspension system are retained, while the damping force is not provided by a conventional passive damper and can be modulated (adjusted) in accordance with the operating conditions by means of an active damper. More recently, the possible application of electrorheological (ER) and magnetorheological (MR) fluids in the development of controllable dampers has attracted considerable interest in this context [5]. Passive suspension systems cannot meet the conflicting requirements of today's high-performance automobiles. Therefore, the need to incorporate active and semi-active suspension systems in automobiles has received much attention [6].

Fuzzy logic control has been used in many applications including cruise control, automatic transmissions, Sendai subway operation, cold-rolling mills, fish processing machine, robots, self-parking car, image stabilizer for video camera, and a fully automated washing machine [7]. Cherry and Jones used a fuzzy logic controller to control a 47-degree-of-freedom (DOF) multibody automotive suspension [8]. By modeling the entire car, the vertical (heave), pitch, and roll responses to selected inputs were analyzed.

Rao et al. [9] considered passive and semi-active control methods for the analysis of an omnibus. Their simulation results indicated a considerable difference between the responses from passive semi-active suspension schemes. Li and Zhao [10] established a mathematical model for a semi-active suspension system based on a quarter vehicle. A fuzzy controller was proposed and its fuzzy rules were deduced using Matlab fuzzy logic control toolbox. The response curves for random road profile excitations of a semi-active suspension system were compared with those of a passive suspension system. The results indicated that the body vertical acceleration was reduced considerably through the use of fuzzy control while the vehicle ride comfort and handling stability were improved as well.

Slaski and Maciejewski [11] presented skyhook-based and fuzzy logic skyhook-based control methods for a semi-active suspension system. Their study confirmed that the method provided better compromise between the body and wheel displacements while giving much more flexibility in the construction of the control strategy than with the classical on-off skyhook control strategy.

Given the proven diversity of fuzzy logic control, this technique is incorporated for control of a semi-active suspension system in the present paper. It is difficult to control the parameters of a fuzzy logic control system because it may not be possible to design the rules without the help of an expert or the process may be rigorous involving a number of iterations. Therefore, a biologically inspired optimization method is utilized in the tuning of the important scaling parameters of the controller. Specifically, particle swarm optimization (PSO) method is used, which gives better results both in terms of convergence and computation time in comparison with genetic algorithms (GA). PSO uses a mathematical model that somewhat mimics a flock of birds in search of food. Every bird is considered a particle and it keeps record of its best position and velocity. The best position and velocity of the complete flock are also recorded. In this manner a bird continuously orients itself in the best path based on its own experience and that of the entire flock. This technique has been widely used in engineering problems [12].

The present paper uses quarter car passive and semi-active suspension systems, which are modeled in Simulink. Fuzzy logic control is used in the semi-active suspension system. The inputs and the output of fuzzy logic controller are normalized and gain factors are incorporated into the system. These gain factors are evaluated by performing off-line tuning using PSO in an optimal manner. Based on the optimized tuning parameters, the maximum output of the damper is determined. Various models are designed based on a variety of control algorithms, input parameters, damping coefficient limits, and membership functions. The models are compared for road handling and ride comfort. Two different types of road disturbance profile are considered in the present paper. The rest of the paper is organized as follows.  Section 2 presents modeling of the systems along with the implementation of the PSO technique.  Section 3 discusses the simulation results.  Section 4 provides concluding remarks on the presented work.

## 2. Modeling of Systems

**Figure 1:**
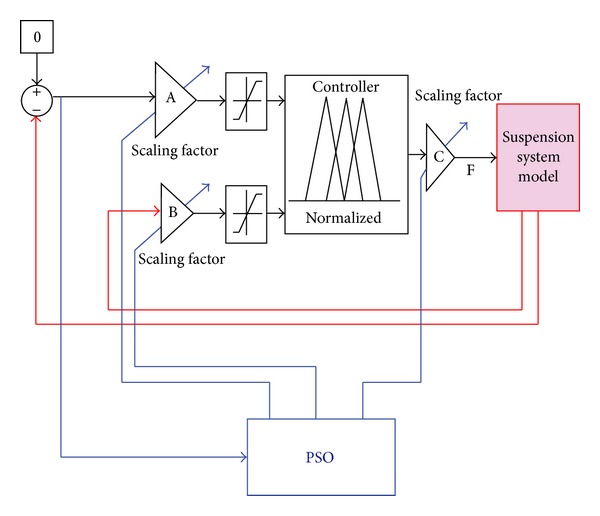
Block diagram of PSO tuned fuzzy logic control system.

This section describes the modeling aspects of the considered systems. The block diagram fuzzy logic control system, which is through PSO, is presented in Figure 1. Two scaling factors, A and B, are incorporated for the inputs of the controller while the scaling factor C is incorporated with the output of the controller. These scaling factors are optimized because otherwise there would be the need to manually adjust the ranges of the fuzzy membership functions of the inputs and the output. Relative displacement of the sprung mass with respect to the road disturbance is fed into the scaling factor A while the relative velocity across the sprung and the unsprung masses is fed to the scaling factor B. The scaling factor C denotes the optimized value of the maximum damping coefficient of the variable damper. The PSO algorithm gets the value of the objective function at each iteration and evaluates the corresponding values of the scaling factors.

### 2.1. Quarter Car Model

**Figure 2:**
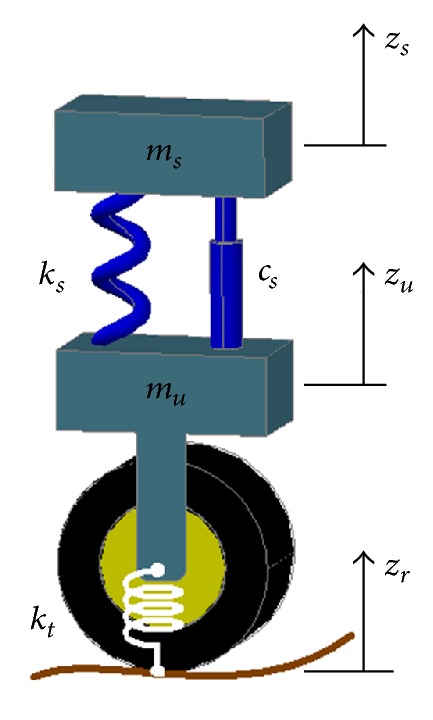
Quarter car model [14].

The quarter car model of a passive suspension system is shown in Figure 2. This model has two degrees of freedom. For simulation purposes, the quarter car parameters are taken from the reference data in [13], which are given in Table 1.

The mathematical model of the passive suspension system is described by (1) and (2) while that of the semi-active suspension system is given by (3) and (4):

(1) $$\begin{array}{c} m_{s}{\ddot{z}}_{s}=-c_{s}\left({\dot{z}}_{s}-{\dot{z}}_{u}\right)-k_{s}\left(z_{s}-z_{u}\right), \end{array}$$

(2) $$\begin{array}{c} m_{u}{\ddot{z}}_{u}=c_{s}\left({\dot{z}}_{s}-{\dot{z}}_{u}\right)+k_{s}\left(z_{s}-z_{u}\right)-k_{t}\left(z_{u}-z_{r}\right), \end{array}$$

(3) $$\begin{array}{c} m_{s}{\ddot{z}}_{s}=-c_{s}\left({\dot{z}}_{s}-{\dot{z}}_{u}\right)-k_{s}\left(z_{s}-z_{u}\right)-c_{a}\left({\dot{z}}_{s}-{\dot{z}}_{u}\right), \end{array}$$

(4) $$\begin{array}{c} m_{u}{\ddot{z}}_{u}=\left(c_{s}+c_{a}\right)\left({\dot{z}}_{s}-{\dot{z}}_{u}\right)+k_{s}\left(z_{s}-z_{u}\right)-k_{t}\left(z_{u}-z_{r}\right). \end{array}$$

Here, *m*_*s*_ denotes the sprung mass, *m*_*u*_ denotes the unsprung mass, *c*_*a*_ is the adaptable damping coefficient of suspension, *c*_*s*_ is the fixed damping coefficient that is used in both passive and semi-active suspensions, *k*_*s*_ and *k*_*t*_ are the spring coefficients of the suspension and the tire, respectively, *z*_*s*_ is the displacement of the sprung mass, *z*_*u*_ is the displacement of the unsprung mass, and *z*_*r*_ is the road input disturbance.

**Figure 3:**
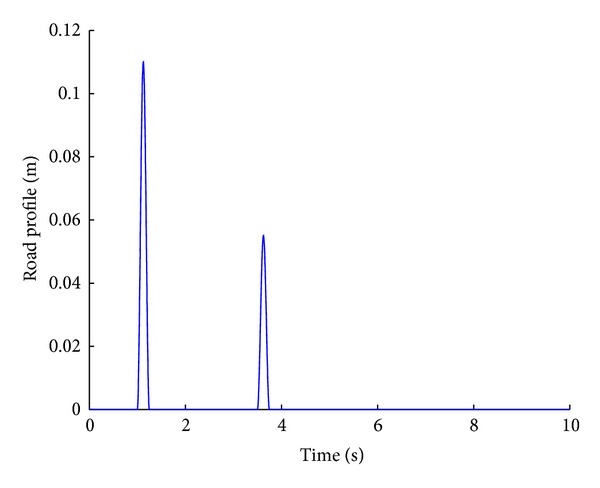
Sinusoidal road profile.

In order to carry out a comparison between the passive and the fuzzy-logic-based semi-active suspension systems, two types of road profiles are considered. The sinusoidal profile is given by (5) and shown in Figure 3:

(5) $$\begin{array}{c} z_{r}=\frac{a}{2}\left\{1-\cos\left(8\pi t\right)\right\}, \end{array}$$

where

(6) $$\begin{array}{c} a=\{\begin{array}{cc} 0.11\text{ m} & \text{for}\;1\leq t\leq 1.25\\ 0.05\text{ m} & \text{for}\;3.5\leq t\leq 3.75\\ 0 & \text{otherwise}. \end{array} \end{array}$$

The road disturbance is a combination of two different sinusoidal functions and hence the performance of the suspension systems can be clearly predicted for a variety of road profiles.

**Figure 4:**
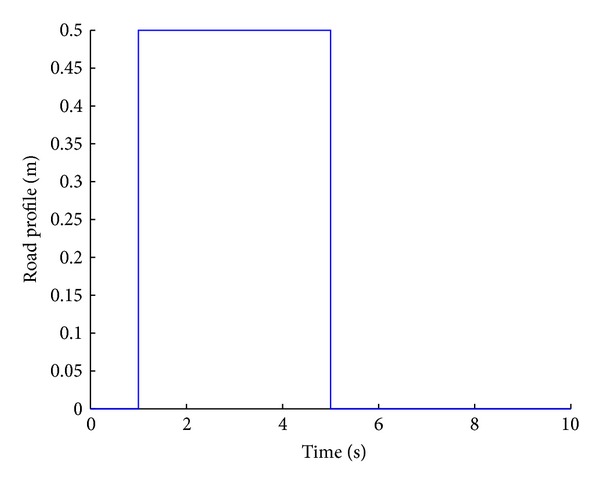
Step road profile.

A step input of 0.5 m step height is considered as shown in Figure 4. The step road profile is a combination of two step disturbances: a step up and a step down. The performance of systems can be validated on a broader perspective.

For a semi-active suspension model, the damping coefficient needs to be varied. In order to incorporate variability of the damping coefficient, a fuzzy logic controller is included in the design scheme.

### 2.2. Optimized Fuzzy Logic Controller

**Figure 5:**
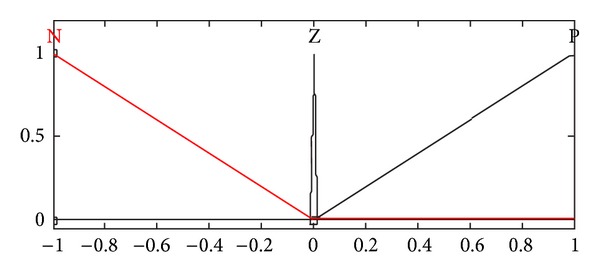
Membership function of relative velocity.

**Figure 6:**
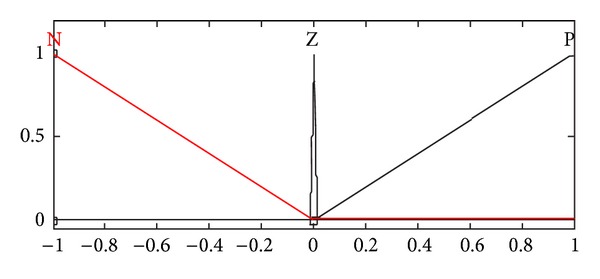
Membership function of relative displacement.

**Figure 7:**
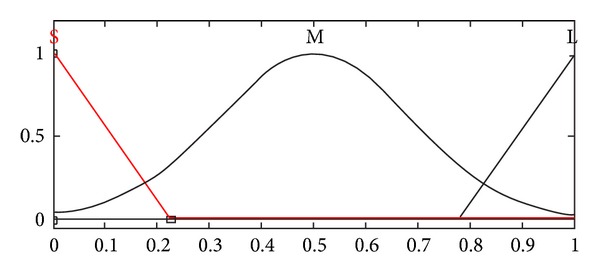
Membership function of damping coefficient.

A fuzzy logic controller involves fuzzification interface, fuzzy rule base, decision making logic, and defuzzification interface [7, 15]. In fuzzification, crisp values are transformed into linguistic variables that are further processed using a fuzzy rule base, in the decision making process—the compositional rule of inference. Finally, linguistic variables are transformed back to crisp values through defuzzification for use by the physical plant (suspension system). In the present paper, the optimized fuzzy logic controller comprises two inputs, namely, relative displacement and relative velocity, while the damping coefficient is the resulting output that eventually controls the response of the vehicle. Each input has the three membership functions: N (triangular), Z (singleton), and P (triangular), as shown in Figures 5 and 6, while the output comprises three membership functions: S (triangular), M (Gaussian), and L (triangular), as shown in Figure 7. All negative values are grouped in membership function “N” while the positive ones are represented by “P.” The domains of small, medium, and higher damping coefficient values are represented by the membership functions, namely, S, M, and L respectively.

Based on the three membership functions for each input, a maximum of nine rules may be formulated. These rules are given in Table 2. All the input and output variables have been normalized; therefore, the universe of discourse for each input is [−1,1] while that for the output is [0,1]. Mamdani inference system is selected while using the “and” operator to represent the minimum function and the centroid method for defuzzification [7, 15]. The rules are formulated so as to retain the optimized combination of performance parameters for both ride comfort and road handling.

### 2.3. Particle Swarm Optimization (PSO)

PSO is inspired by the behavior exhibited by flocks of birds and schools of fish. The main approach of this algorithm is to search through an n-dimensional problem to optimize an objective function. The PSO technique was selected because its implementation is relatively simple and the convergence behavior is good. Furthermore, it has the ability to reach the global optimum while avoiding local optima [16].

**Figure 8:**
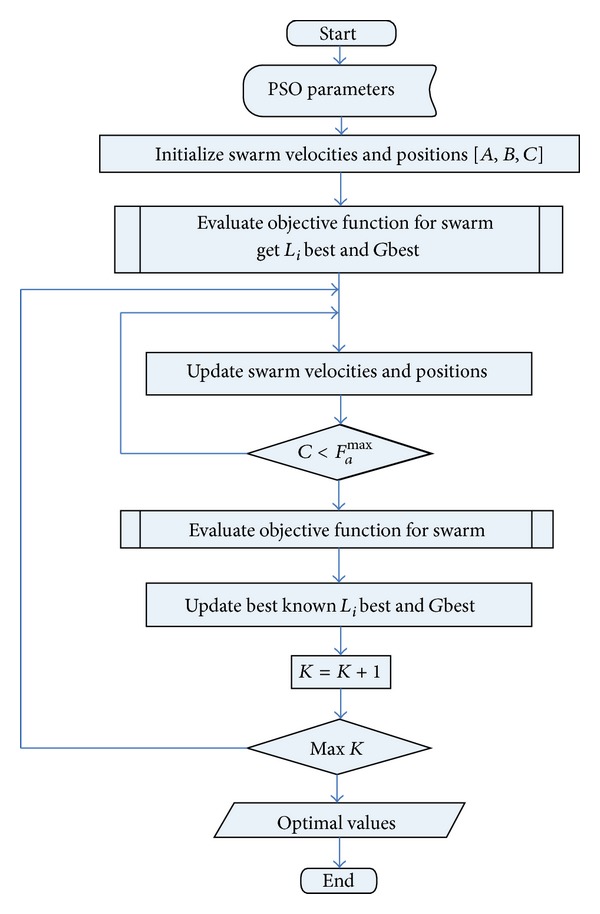
Flow chart of PSO algorithm [17].

The swarm comprises a fixed number of particles. These particles collaboratively search for an optimal position. A cognitive component encourages the particles to improve upon their individual best position thus far, while a social component always pulls the particles toward the global best position thus far. A flow chart of the algorithm is given in Figure 8. The PSO parameters are the constant values that are used in the evaluation of the position and the velocity of each particle. Swarm positions and velocities are initialized for each particle.

The objective function used in the present application is the suspension displacement with respect to the step road disturbance, which needs to be minimized. The best known position for each particle is tracked and memorized as *L*_*i*_best, while the corresponding best known position for the complete swarm is denoted by *G*best. In the *k* + 1th iteration, the position of each particle (*p*_*i*_) is updated by velocity (*v*_*i*_) as given by

(7) vi(k+1)=w∗vi(k)+c1∗r1(k)∗(Libest−pi(k)) +c2∗r2(k)∗(Gbest−pi(k)),pi(k+1)=pi(k)+vi(k).

Here, *c*_1_ and *c*_2_ denote cognitive and social accelerations for the local best position and the global best position, respectively, *w* is the inertial weight constant, and *r*_1_(*k*) and *r*_2_(*k*) represent random numbers generated in the uniform distribution interval of [0,1]. Each particle optimizes a set of three variables *A*, *B*, and *C*. *F*_*a*_^max^ indicates the maximum damping coefficient limit of the available damper. The algorithm keeps on evaluating the optimized values of the desired parameters until the final value of *C* reaches *F*_*a*_^max^ or the number of iterations reaches its limit.

## 3. Simulation Results and Discussion

The proposed approach is implemented on the suspension system of a quarter-vehicle model, and computer simulations are carried out. This section describes this simulation exercise and analyzes the obtained results. Modeling and simulation are performed in Matlab 7.8.0 (R2009a). The fuzzy logic controller is designed using the Matlab Fuzzy Tool Box while simulations are performed in Simulink. The PSO algorithm is programmed in Matlab and executed to determine the three optimized gain parameters of the fuzzy logic controller. The reference algorithm is employed on an active suspension system while the present work implements the technique on a semi-active suspension system. The parameters used in the PSO algorithm are given in Table 3.

Optimized values obtained through the PSO algorithm for the fuzzy semi-active suspension system are *A* = 19.0129, *B* = 12.3983, and *C* = 3802.3. In order to evaluate the performance of a fuzzy-logic-based semi-active suspension system, several different systems have been developed and simulated, as presented next.

### 3.1. Systems Based on Various Control Algorithms

**Figure 9:**
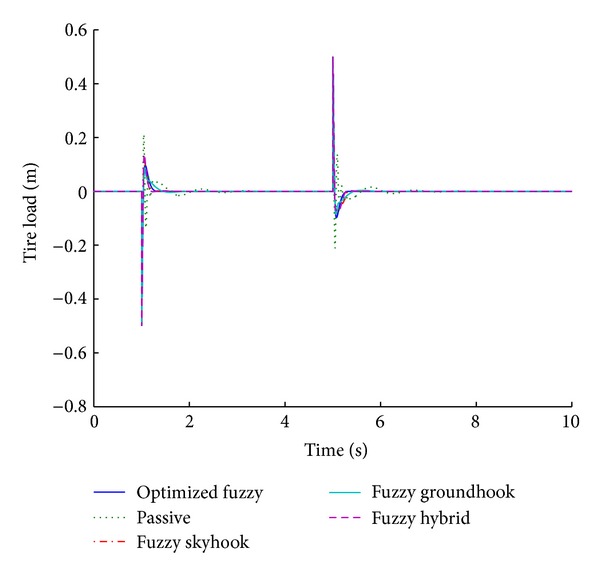
Tire load for various control algorithms (step input).

**Figure 10:**
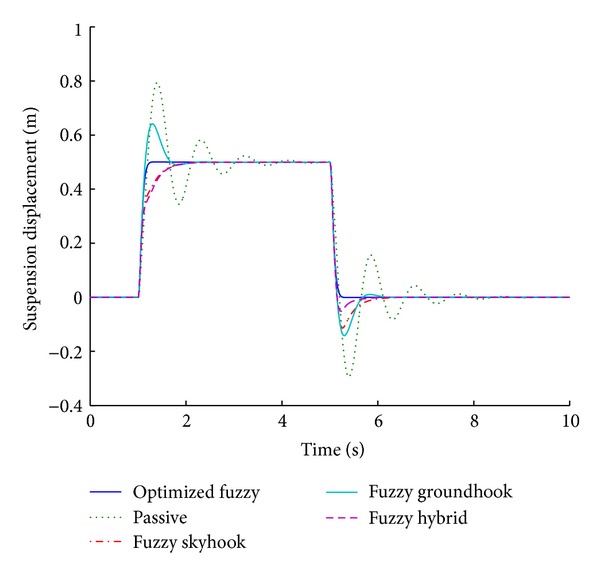
Suspension displacement for various control algorithms (step input).

Fuzzy skyhook system is designed using the theoretical background of skyhook system in which the relative velocity across the two masses and the absolute velocity of the sprung mass are monitored in order to modulate the damping value. In case of the fuzzy groundhook system, the absolute velocity of the sprung mass is replaced by that of the unsprung mass. The fuzzy hybrid system combines the strategy of fuzzy groundhook and skyhook systems. Figures 9 and 10 present the performance results for various control algorithms in response to step road disturbance.

Values of important performance parameters in different control algorithms are tabulated in Table 4 with respect to a step road disturbance.

The bold values indicate percentage overshoot while the italic values indicate the stabilizing time expressed in seconds. The same format is maintained throughout the tabular presentation of the performance parameters. The top two values in each grid correspond to the initial disturbance and the bottom ones correspond to the second part of the disturbance. Suspension displacement is a good indicator of the ride comfort while tire load is a measure of vehicle handling.

For a step road disturbance, the optimized fuzzy system outperforms all other systems, with regard to suspension displacement. There is no overshoot and the system gets stabilized at the earliest in relation to other systems in the comparison. It follows that the optimized fuzzy system gives the best ride comfort among all the control algorithms. The passive system does not stabilize at all and it gives the highest values of overshoot for the suspension displacement. Fuzzy hybrid approach combines the strategies of skyhook and groundhook. Therefore, it is better than each of the other control algorithms.

With regard to the tire load, the shortest stabilizing time periods are obtained by the optimized fuzzy logic system while groundhook offers the minimum percentage overshoot. However, the optimized fuzzy system is still comparable with the groundhook system, with regard to vehicle handling.

**Figure 11:**
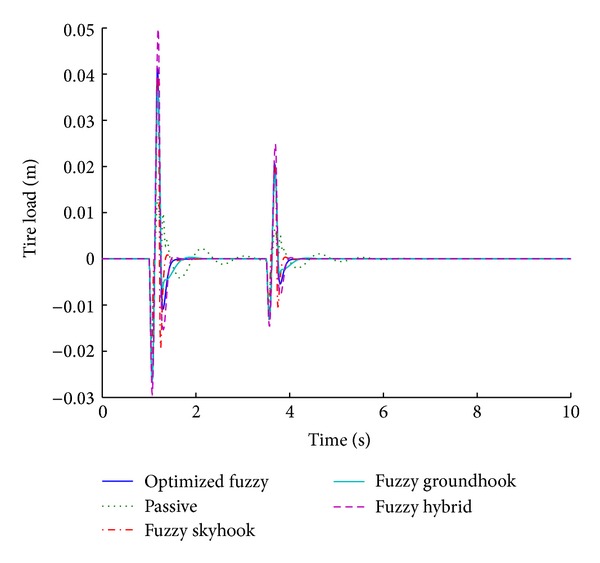
Tire load for various control algorithms (sinusoidal input).

**Figure 12:**
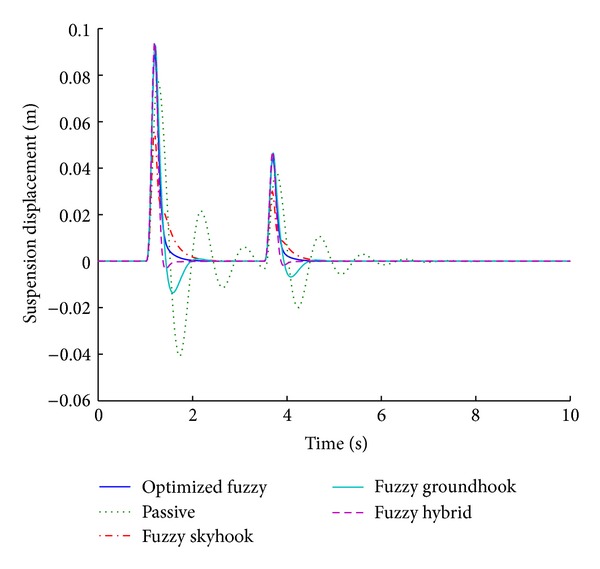
Suspension displacement for various control algorithms (sinusoidal input).

Figures 11 and 12 present the performance results of various systems based on the used control algorithms, in response to a sinusoidal road disturbance.

The values of important performance parameters for various control algorithms are given in Table 5, corresponding to a sinusoidal road disturbance.

For a sinusoidal road disturbance, the optimized fuzzy system still provides the best performance by giving a zero overshoot for suspension displacement. However, the fuzzy hybrid gives the shortest stabilizing time periods. It is worth mentioning that the optimized fuzzy system performs better even for sinusoidal input, thereby validating its performance for different inputs.

### 3.2. Systems Based on Various Input Parameters

Various systems are modeled on the basis of different combinations of input parameters of the fuzzy logic controller. Two different systems are modeled on the basis of relative velocity and relative displacement across the sprung mass and the unsprung mass. While the fuzzy input variables having three membership functions each give rise to a total of nine rules, the selection of two membership functions each leads to a total of four rules, as given in Table 6. The membership functions represented by letters follow the same convention as described earlier in Table 2. Other systems incorporate the combinational parametric schemes of absolute velocity and absolute acceleration of the sprung mass.

**Figure 13:**
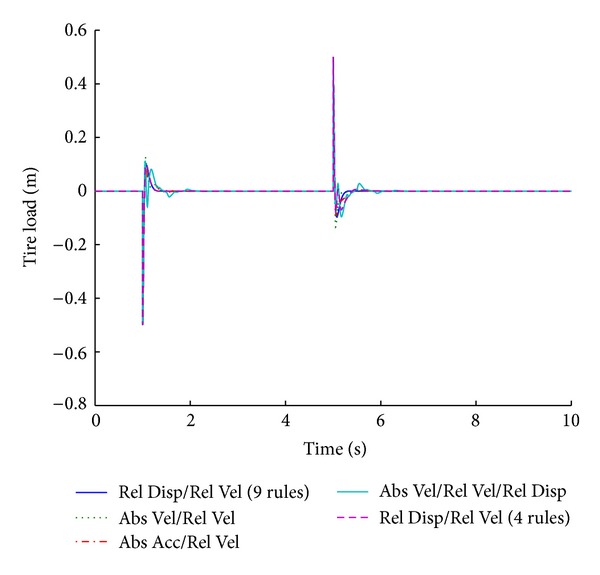
Tire load for various input parameters (step input).

**Figure 14:**
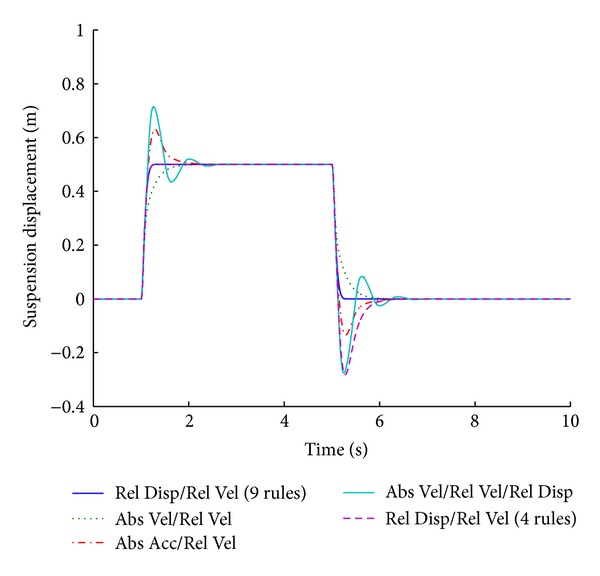
Suspension displacement for various input parameters (step input).

Figures 13 and 14 show the performance results of various systems based on specific input parameters, in response to a step road disturbance. Values of important performance parameters of the systems, based on various input parameters, are given in Table 7, for a step road disturbance.

Various systems are designed based on the input parameters of the fuzzy logic controllers. The fuzzy systems that incorporate fuzzy inputs of relative displacement and relative velocity provide the best results for suspension displacement in response to a step road disturbance. This system is governed by nine rules (Table 2). The fuzzy system based on the same input parameters having four rules (Table 6) performs in the same way for the first part of the disturbance, but its performance deteriorates in the second part. The system based on relative and absolute velocities also does not generate any overshoot but it gives inferior results with regard to the stabilizing time period.

**Figure 15:**
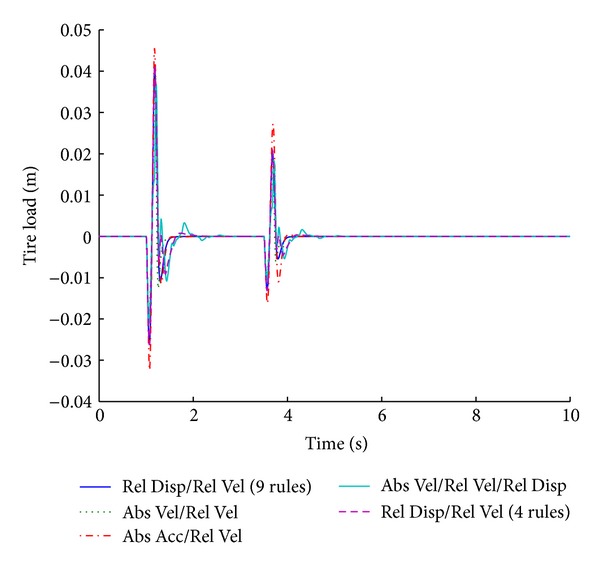
Tire load for various input parameters (sinusoidal input).

**Figure 16:**
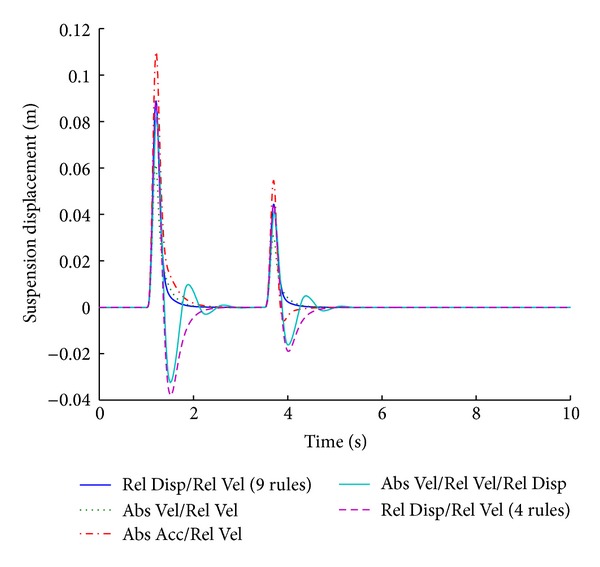
Suspension displacement for various input parameters (sinusoidal input).

The optimized fuzzy system gives better performance for the vehicle handling parameter of tire load while maintaining the combined factors of minimum overshoot and shortest stabilizing time. Figures 15 and 16 show the performance results of various systems based on specific input parameters in response to a sinusoidal road disturbance.

Values of important performance parameters of the systems based on various input parameters are given in Table 8, in response to a sinusoidal road disturbance.

The same fuzzy system outperforms all other systems even for sinusoidal road disturbance.

### 3.3. Systems Based on Variable Payload

**Figure 17:**
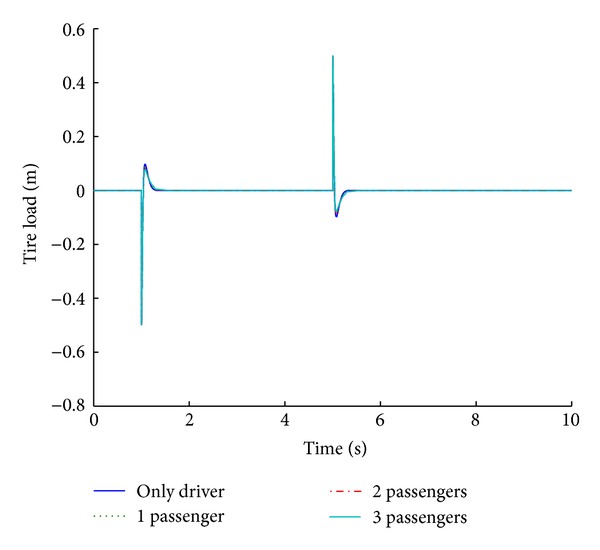
Tire load for payload variation (step input).

**Figure 18:**
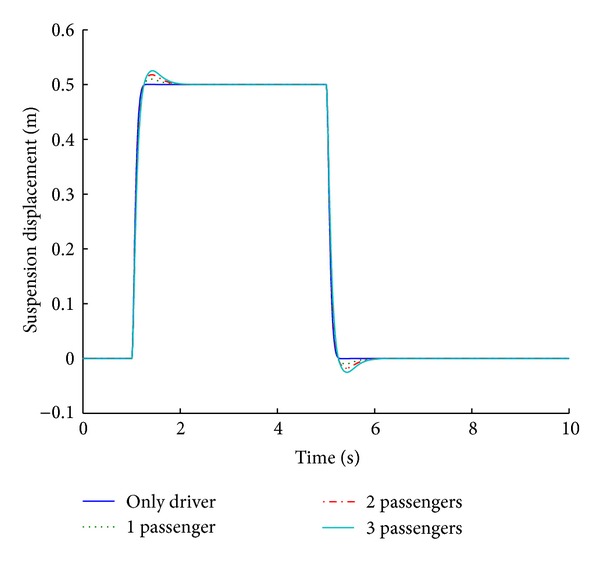
Suspension displacement for payload variation (step input).

Various systems are modeled and simulated for analyzing the impact of payload variation on the performance parameters. The weight of each passenger is taken as 100 kg, which transforms to 25 kg in the quarter car parameter. Figures 17 and 18 show the performance results of various systems based on variable payloads in response to a step road disturbance. Values of important performance parameters of the systems for various payloads are given in Table 9 in response to a step road disturbance.

**Figure 19:**
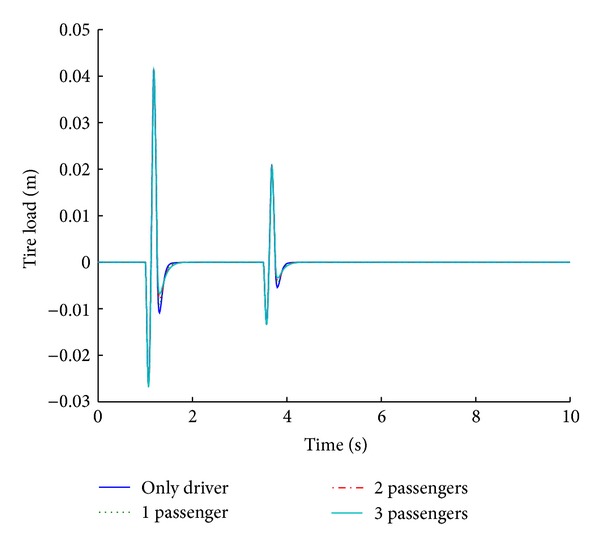
Tire load for payload variation (sinusoidal input).

**Figure 20:**
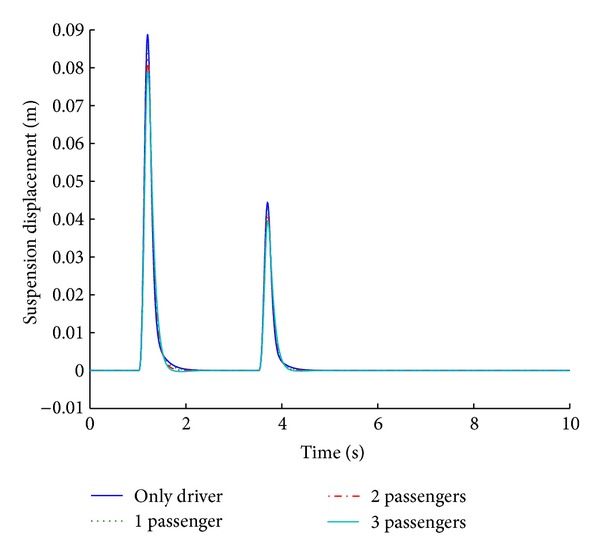
Suspension displacement for payload variation (sinusoidal input).

The optimized fuzzy system is analyzed for its performance under conditions of variable payload. For a step road disturbance, there is a slight variation in the ride comfort in relation to the number of passengers, while vehicle handling improves with the increase in the number of passengers. Figures 19 and 20 show the performance results of various systems under variable payload, in response to a sinusoidal road disturbance.

Values of important performance parameters of the systems for various payloads are given in Table 10 in response to a sinusoidal road disturbance.

For a sinusoidal road disturbance, both the ride comfort and the vehicle handling characteristics show improvement with the increase in the number of passengers. Therefore, the designed controller provides optimum results even with a variable payload.

### 3.4. Systems Based on Variable Damping Coefficient Limits

**Figure 21:**
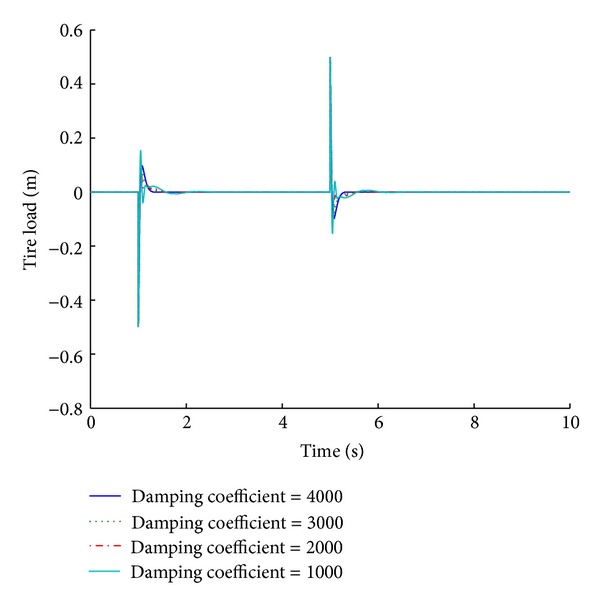
Tire load for maximum damping coefficient limit (step input).

**Figure 22:**
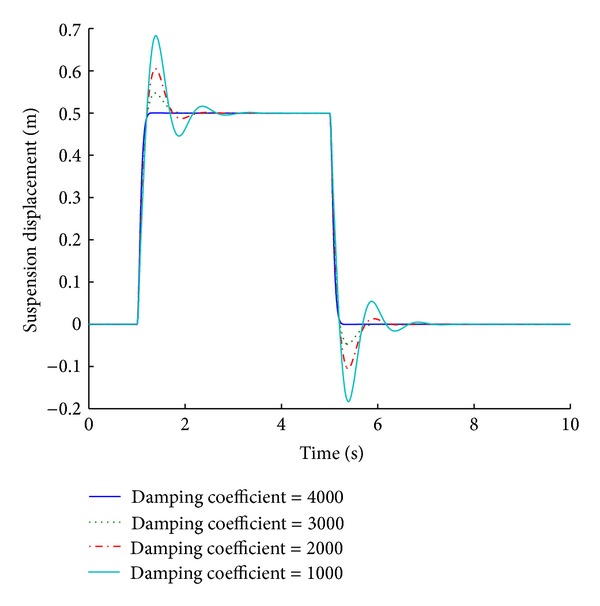
Suspension displacement for maximum damping coefficient limit (step input).

Using the optimized value of damping coefficient, four systems are designed with variable damping coefficient limits of 1000, 2000, 3000, and 4000 N s/m. Simulation of these systems presents an insight into the parametric analysis of the variable damping coefficient limits. Figures 21 and 22 depict the performance results of various systems based on the maximum damping coefficient limit in response to a step road disturbance.

Values of some important performance parameters of the systems based on maximum damping coefficient limit are given in Table 11 in response to a step road disturbance.

**Figure 23:**
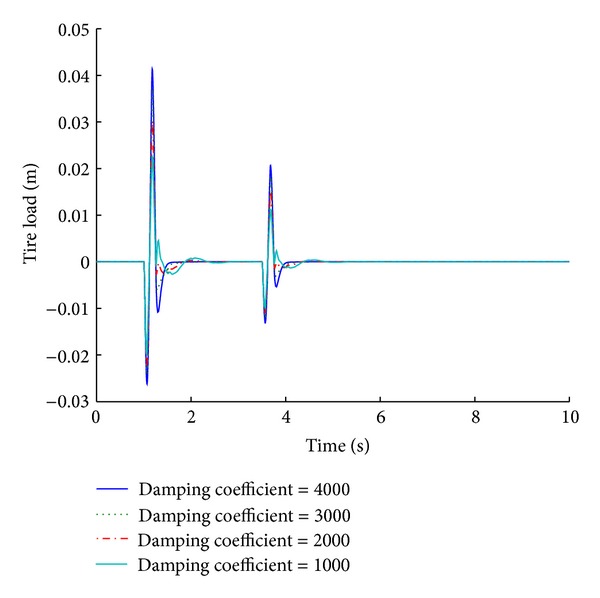
Tire load for maximum damping coefficient limit (sinusoidal input).

**Figure 24:**
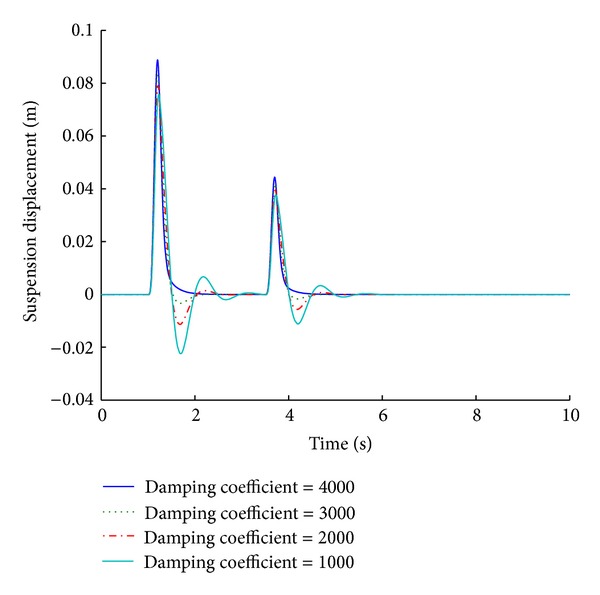
Suspension displacement for maximum damping coefficient limit (sinusoidal input).

Selection of an appropriate damping coefficient limit is very significant performance consideration. Systems based on different damping coefficient limits exhibit different results as is evident from the tabulated values. A damping coefficient limit of 4000 N s/m (the optimized value is 3802.3) gives the best results for ride comfort and road handling under a step road disturbance. The performance deteriorates as the damping value decreases. Figures 23 and 24 give the performance results of various systems for a maximum damping coefficient limit, in response to a sinusoidal road disturbance.

Values of important performance parameters of systems based on maximum damping coefficient limit are given in Table 12 in response to a sinusoidal road disturbance.

For a sinusoidal road disturbance, a damping coefficient of 1000 N s/m yields a nonstabilizing behavior of the system with regard to the ride comfort parameters. A damping coefficient of 4000 N s/m yields the best results for suspension displacement, and with regard to the tire load it gives the shortest stabilizing time and comparable overshoot values.

## 4. Conclusions

This paper presented a successful application of a hybrid fuzzy-logic technique in the design of a semi-active suspension system for an automobile. The relative displacement and the relative velocity of the suspension were the inputs to the fuzzy logic controller. The input and output membership functions of the fuzzy system were normalized using scaling factors. The incorporation of particle swarm optimization (PSO) to tune the controller parameters is a novel approach in the context of semi-active suspensions. Specifically, PSO performed off-line tuning of the gain factors of the fuzzy logic controller of a quarter car suspension model. The PSO objective function was selected as the offset of the suspension displacement and the road disturbance, which was minimized. The developed algorithm generated a damping coefficient limit for the damper of the suspension system. The performance of the fuzzy logic controlled system was found to be much better than that of the passive system in terms of both road handling and ride comfort. Furthermore, by comparing the performance under different control algorithms, it was found that the optimized fuzzy logic system was superior. The designed semi-active system performed better within the entire range of payload variation. Furthermore, the fuzzy heuristic rules coped well with the changing disturbances.

## Figures and Tables

**Table 1:** Quarter car parameters.

Parameter	Symbol	Value	Units
Sprung mass	*m*_*s*_	300	kg
Unsprung mass	*m*_*u*_	36	kg
Sprung mass damping coefficient	*c*_*s*_	1000	N s/m
Spring stiffness	*k*_*s*_	16000	N/m
Tire stiffness	*k*_*t*_	160000	N/m

**Table 2:** Fuzzy rule base (nine rules).

		Relative displacement		
		N	Z	P
Relative velocity	N	M	L	L
	Z	S	S	S
	P	L	L	M

**Table 3:** Parameters of the PSO algorithm.

Parameter	Symbol
Swarm size	30
Number of iterations	30
Unknown variables	3
Cognitive acceleration	1.2
Social acceleration	1.6
Inertial weight	0.4

**Table 4:** Performance comparison of various control techniques for step road profile.

Parameters	Control algorithms									
	Optimized fuzzy		Passive		Fuzzy skyhook		Fuzzy groundhook		Fuzzy hybrid	
Tire load	**10**	*1.3 *	**21**	—	**11**	*1.5 *	**8**	*1.9 *	**14**	*1.6 *
	−**10**	*5.3 *	−**21**	—	−**9**	*5.6 *	−**9**	*5.9 *	−**7**	*5.5 *
Suspension displacement	**0**	*1.3 *	**60**	—	**0**	*2.3 *	**28**	*2.5 *	**0**	*2.4 *
	**0**	*5.3 *	−**30**	—	−**12**	*6.4 *	−**14**	*6.5 *	−**5**	*6.1 *

Bold cells give percentage overshoot.

Italic cells give stabilizing time in seconds.

**Table 5:** Performance comparison of various control techniques for sinusoidal road profile.

Parameters	Control algorithms									
	Optimized fuzzy		Passive		Fuzzy skyhook		Fuzzy groundhook		Fuzzy hybrid	
Tire load	−**1**	*1.6 *	**1**	—	−**1.9**	*1.8 *	−**0.6**	*2.1 *	−**2**	*1.6 *
	−**0.5**	*4 *	**0.5**	*6.4 *	−**1**	*4 *	−**0.3**	*4.5 *	−**1**	*4.1 *
Suspension displacement	**0**	*2.2 *	−**4**	—	**0**	*2.7 *	−**1.4**	*2.4 *	−**0.3**	*1.7 *
	**0**	*4.5 *	−**2**	*7.5 *	**0**	*5 *	−**0.7**	*4.8 *	−**0.2**	*4.1 *

Bold cells give percentage overshoot.

Italic cells give stabilizing time in seconds.

**Table 6:** Fuzzy rule base (four rules).

		Relative displacement	
		N	P
Relative velocity	N	M	S
	P	L	M

**Table 7:** Performance comparison of various input parameters in fuzzy logic control for step road profile.

Parameters	Input parameters for fuzzy logic controller									
	Relative velocity/ relative displacement (9 rules)		Absolute velocity/ relative velocity		Absolute acceleration/ relative velocity		Absolute velocity/ relative velocity/ relative displacement		Relative velocity/ relative displacement (4 rules)	
Tire load	**9.7**	*1.3 *	**13**	*1.2 *	**9**	*1.7 *	**11.2**	*2.2 *	**9.7**	*1.3 *
	−**9.7**	*5.3 *	−**13**	*5.2 *	−**9**	*5.7 *	−**9.6**	*6.2 *	−**8**	*5.8 *
Suspension displacement	**0**	*1.3 *	**0**	*2.1 *	**26**	*2.5 *	**43**	*3 *	**0**	*1.3 *
	**0**	*5.3 *	**0**	*6.1 *	−**13**	*6.5 *	−**28**	*7 *	−**28**	*6.4 *

Bold cells give percentage overshoot.

Italic cells give stabilizing time in seconds.

**Table 8:** Performance comparison of various input parameters in fuzzy logic control for sinusoidal road profile.

Parameters	Input parameters for fuzzy logic controller									
	Relative velocity/ relative displacement (9 rules)		Absolute velocity/ relative velocity		Absolute acceleration/ relative velocity		Absolute velocity/ relative velocity/ relative displacement		Relative velocity/ relative displacement (4 rules)	
Tire load	−**1.1**	*1.6 *	−**1**	*1.7 *	−**1.1**	*1.6 *	−**1.1**	*2.7 *	−**0.9**	*2.1 *
	−**0.5**	*4.1 *	−**0.6**	*4.1 *	−**1.1**	*4.2 *	−**0.5**	*4.8 *	−**0.4**	*4.4 *
Suspension displacement	**0**	*2.2 *	**0**	*2.3 *	**0**	*2.5 *	−**3.2**	*3 *	−**4**	*2.6 *
	**0**	*4.6 *	**0**	*4.6 *	−**0.6**	*4.5 *	−**1.6**	*5.4 *	−**2**	*4.9 *

Bold cells give percentage overshoot.

Italic cells give stabilizing time in seconds.

**Table 9:** Performance comparison of systems based on different payloads for step road profile.

Parameters	Variable payload (number of passengers)							
	Only driver		1		2		3	
Tire load	**9.7**	*1.3 *	**9**	*1.4 *	**8.3**	*1.5 *	**7.7**	*1.5 *
	−**9.7**	*5.3 *	−**9**	*5.4 *	−**8.3**	*5.5 *	−**7.7**	*5.5 *
Suspension displacement	**0**	*1.3 *	**2**	*1.9 *	**3.6**	*2 *	**5**	*2 *
	**0**	*5.3 *	−**1**	*5.9 *	−**1.8**	*6 *	−**2.5**	*6 *

Bold cells give percentage overshoot.

Italic cells give stabilizing time in seconds.

**Table 10:** Performance comparison of systems based on different payloads for sinusoidal road profile.

Parameters	Variable payload (number of passengers)							
	Only driver		1		2		3	
Tire load	−**1.1**	*1.6 *	−**0.93**	*1.7 *	−**0.8**	*1.7 *	−**0.68**	*1.7 *
	−**0.54**	*4 *	−**0.46**	*4.1 *	−**0.4**	*4.1 *	−**0.34**	*4.2 *
Suspension displacement	**0**	*2.2 *	**0**	*2.1 *	**0**	*1.8 *	**0**	*1.7 *
	**0**	*4.5 *	**0**	*4.4 *	**0**	*4.3 *	**0**	*4.2 *

Bold cells give percentage overshoot.

Italic cells give stabilizing time in seconds.

**Table 11:** Performance comparison of systems based on maximum damping coefficient for step road profile.

Parameters	Maximum damping coefficient limit (N s/m)							
	4000		3000		2000		1000	
Tire load	**9.7**	*1.3 *	**9.6**	*1.5 *	**11.6**	*2 *	**15.4**	*2.5 *
	−**9.7**	*5.3 *	−**9.6**	*5.5 *	−**11.6**	*6 *	−**15.4**	*6.5 *
Suspension displacement	**0**	*1.3 *	**9.6**	*1.9 *	**21**	*2.3 *	**36.6**	*3.5 *
	**0**	*5.3 *	−**4.8**	*5.9 *	−**10.5**	*6.3 *	−**18.3**	*7.5 *

Bold cells give percentage overshoot.

Italic cells give stabilizing time in seconds.

**Table 12:** Performance comparison of systems based on maximum damping coefficient for sinusoidal road profile.

Parameters	Maximum damping coefficient limit (N s/m)							
	4000		3000		2000		1000	
Tire load	−**1.1**	*1.6 *	−**0.61**	*1.8 *	−**0.26**	*2.4 *	−**0.27**	*2.8 *
	−**0.54**	*4.1 *	−**0.31**	*4.2 *	−**0.13**	*4.6 *	−**0.13**	*5.3 *
Suspension displacement	**0**	*2.2 *	−**0.34**	*2.2 *	−**1.1**	*2.9 *	−**2.2**	—
	**0**	*4.6 *	−**0.17**	*4.6 *	−**0.57**	*5 *	−**1.1**	*6 *

Bold cells give percentage overshoot.

Italic cells give stabilizing time in seconds.
